# A retrospective study of californium-252 neutron brachytherapy combined with EBRT versus 3D-CRT in the treatment of esophageal squamous cell cancer

**DOI:** 10.1186/s13014-015-0520-7

**Published:** 2015-10-24

**Authors:** Qifeng Wang, Tao Li, Jinyi Lang, Jie Wang, Jian Wang, Huiming Liu, Xitang Jia, Bo Liu, C-K Chris Wang

**Affiliations:** Department of Radiation Oncology, Sichuan Cancer Hospital, Chengdu, Sichuan 610041 P.R. China; Department of Radiation Oncology, Changzhi Cancer Hospital, Changzhi, Shanxi 046000 P.R. China; Medical Physics Program, School of Mechanical Engineering, Georgia Institute of Technology, Atlanta, GA 30332-0745 USA

**Keywords:** Esophageal squamous cell carcinoma (ESCC), Neutron brachytherapy (NBT), High-LET, Relative biological effectiveness (RBE)

## Abstract

**Background:**

We conducted a retrospective analysis on 884 patients who were diagnosed with esophageal squamous cell carcinoma (ESCC) and treated with either the neutron brachytherapy in combination with external beam radiotherapy (NBT + EBRT) or 3-dimensional conformal radiation therapy (3D-CRT) to determine the differences in efficacy and morbidity between the two treatment groups.

**Methods:**

The 884 ESCC patients treated with either NBT + EBRT or 3D-CRT between 2002 and 2012 were retrospectively reviewed and analyzed. Multivariable Cox regression was used to compare oncologic outcomes of the two groups of patients in the context of other clinically relevant variables. The acute and chronic toxicities associated with the two groups were compared using Fisher exact and log-rank tests, respectively.

**Results:**

Among the 884 patients, 545 received NBT + EBRT and 339 received 3D-CRT (i.e. EBRT-only). The age range is 39–95 years (median 66). The follow-up time range is 3–145 months (median 32). The analysis shows that the NBT + EBRT group has higher overall survival rate and local control rate than that of the 3D-CRT group. The acute toxicity effects were acceptable for both groups of patients with the NBT + EBRT group showing higher rates of leukopenia and thrombocytopenia and the 3D-CRT group showing higher rates on fistula and massive bleeding.

**Conclusions:**

The patients treated with NBT + EBRT showed better oncologic outcomes than those treated with 3D-CRT. The toxicity effects were acceptable for both groups with the NBT + EBRT group showing higher rates on the acute effects and the 3D-CRT group showing higher rates on the late effects.

## Background

Worldwide, an estimated 482,000 new esophageal cancer cases were diagnosed and approximately 407,000 deaths occurred in 2008 [1]. The management of localized esophageal cancers has shifted from surgery or radiation single modality approaches to the trimodality. The current trimodality approach combining chemotherapy, radiation therapy, and surgery, has shown improved survival rates [2]. However, there are patients that either cannot tolerate or decide not to undergo surgery. For these individuals, concurrent chemoradiotherapy (CCRT) is the standard approach.

In recent years, two radiotherapy modalities have become widely used in China to treat esophageal cancers: the neutron brachytherapy in combination with conventional external beam radiotherapy (NBT + EBRT) and the 3-dimensional conformal radiotherapy (3D-CRT). Currently there are no prospective, randomized trials comparing the efficacy of the two modalities for the treatment of esophageal cancer. The choice between the two modalities is not trivial, and their characteristics can have bearing on both disease control and adverse effects [3]. Generally speaking, 3D-CRT tends to treat lymph nodes adjacent to the target with relatively high doses, whereas EBRT tends to cover farther regional nodes with lower doses [4, 5]. Because the radiation dose of NBT is highly localized, the NBT + EBRT has a benefit of sculpting away high-dose regions from normal tissues, e.g. the heart and lungs. In addition to clinical considerations, there are also substantial differences between NBT + EBRT and 3D-CRT (i.e. EBRT-only) in terms of the allocations of time, labor, and health care costs. Accordingly, we conducted a retrospective study on patients of esophageal squamous cell carcinoma (ESCC) who were treated with either NBT + EBRT or 3D-CRT to determine the differences in efficacy and morbidity of the two modalities.

## Methods

### Patients

From January 2002 to November 2012, two groups of patients diagnosed with localized ESCC were treated separately at two different hospitals and with two different radiotherapy modalities. The first group of 545 patients were treated at the Changzhi Cancer Hospital with NBT + EBRT, and the second group of 339 patients were treated at the Sichuan cancer hospital with 3D-CRT (i.e. EBRT-only). All 884 patients had histologically confirmed ESCC measuring 10 cm or less in length. The primary tumors of the patients were limited to the esophagus, with or without the presence of involved regional lymph nodes, i.e., T2-4, N0-1, M0 according to the 2002 American Joint Committee on Cancer (AJCC) clinical staging system. All of the patients had good performance status (i.e. being able to care for himself or herself) as well as adequate hepatic, renal, and hematologic functions. All patients gave their informed consents before treatment in accordance with the Declaration of Helsinki. The informed consents were also approved by the respected Ethics Committees of Changzhi Cancer Hospital and Sichuan Cancer Hospital. The detailed demographic data and tumor characteristics of the patients are given in Table 1.

**Table 1:** The characteristics of patients of the two treatment groups (EBRT + NBT and 3D-CRT) and the univariate analysis of the clinical outcome

Characteristics	Total (%)	EBRT + NBT (%)	3D-CRT (%)	*P* value*	5y OS (%)	*P* value	5y LC (%)	*P* value
Gender				<0.0001		0.174		0.312
Male	583 (66.0)	324 (59.4)	259 (76.4)		24.5		44.7	
Female	301 (34.0)	221 (40.6)	80 (23.6)		30.8		51.9	
Age (years)				0.003		0.291		0.591
≤65	426 (48.2)	284 (52.1)	142 (41.9)		29.9		48.3	
>65	458 (51.8)	261 (47.9)	197 (58.1)		23.5		46.4	
Tumor length				0.220		<0.0001		<0.0001
≤5 cm	409 (46.3)	261 (47.9)	148 (43.7)		31.0		52.4	
>5 cm	475 (53.7)	284 (52.1)	191 (56.3)		22.9		42.4	
Tumor location				<0.0001		0.233		0.581
Upper	329 (37.2)	174 (31.9)	155 (45.7)		26.9		49.2	
Middle	442 (50.0)	309 (56.7)	133 (39.2)		27.8		45.6	
Lower	113 (12.8)	62 (11.4)	51 (15.0)		20.9		46.4	
T stage				0.006		<0.0001		<0.0001
T1	32 (3.6)	20 (3.7)	12 (3.5)		64.1		73.1	
T2	163 (18.4)	107 (19.6)	56 (16.5)		32.9		51.7	
T3	305 (34.5)	164 (30.1)	141 (41.6)		31.2		53.9	
T4	384 (43.4)	254 (46.6)	130 (38.3)		17.6		37.7	
N stage				<0.0001				<0.0001
N0	461 (52.1)	338 (62.0)	123 (36.3)		32.6		53.2	
N1	423 (47.9)	207 (38.0)	216 (63.7)		20.2		40.0	
AJCC stage				<0.0001		<0.0001		<0.0001
I	28 (3.2)	20(3.7)	8 (2.4)		72.3		74.5	
IIa	280 (31.7)	195 (35.8)	85 (25.1)		36.0		56.6	
IIb	55 (6.2)	22 (4.0)	33 (9.7)		29.3		35.2	
III	521 (58.9)	308 (56.5)	213 (62.8)		19.2		41.1	
Radiation dose				<0.0001		0.263		0.658
≤60Gy	321 (36.3)	91 (16.7)	230 (67.8)		22.9		44.7	
>60Gy	563 (63.7)	454 (83.3)	109 (32.2)		29.0		48.6	

*Abbreviations*: *EBRT + NBT* external beam radiotherapy plus neutron brachytherapy; *3D-CRT* three dimensional conform radiotherapy

*P* value*: the *P* values corresponding to the log-rank test between the two treatment groups (EBRT + NBT and 3D-CRT)

### Radiotherapy

#### Group 1: NBT + EBRT

**Fig. 1:**
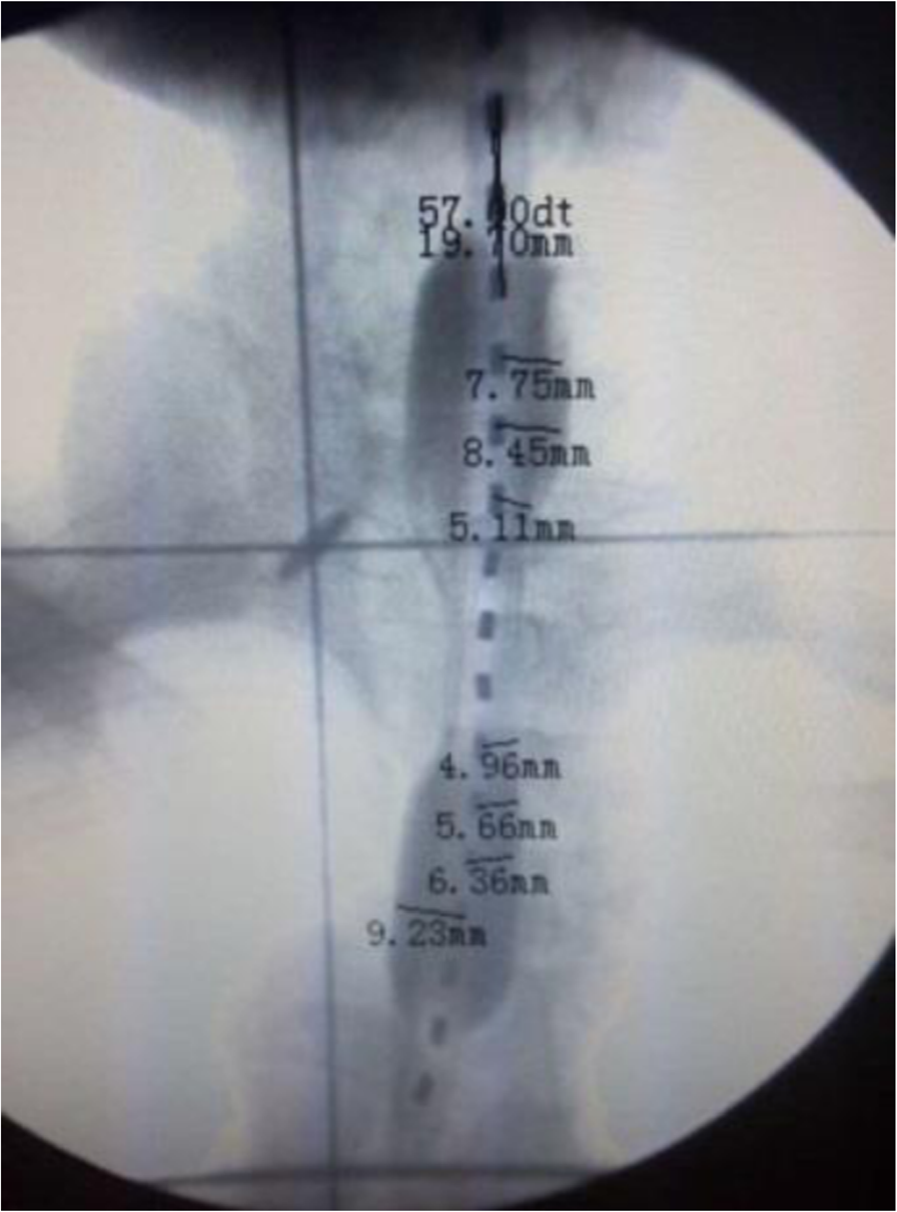
The x-ray image showing the tumor regression conditions before each of the 2–5 treatment fractions of NBT

For the patients treated with NBT + EBRT, NBT and EBRT were interchangeably implemented over the treatment time period. Specifically, patients who were able to take normal food or soft diet underwent one fraction of NBT and five fractions of EBRT per week. A nominal treatment lasted for 5 weeks, and it included five fractions of NBT and 25 fractions of EBRT. Some patients who were only able to take liquid diet were first treated with EBRT-only for 2 or 3 weeks and then followed with NBT + EBRT for the next 2 or 3 weeks. Since these patients only received two or three fractions of NBT, the dose for each NBT fraction was adjusted accordingly [4]. The EBRT was carried out by a 6 MV linear accelerator. The treatment field size was determined according to the CT and barium swallow test results. Two-field technique (one anterior field and one posterior field) or three-field technique (one anterior field and two posterior fields) was used to treat the upper segment of the esophagus. Only the three-field technique (one anterior field and two posterior fields) was used to treat the middle and lower segments of the esophagus. The upper and lower boundaries of the treatment field were determined by adding 3–5 cm from the visible disease area shown on the CT/barium swallow images. In general, the width of the treatment field was 6–7 cm, and the total length was approximately 15 cm. The EBRT follows the normal fractionation with five fractions per week, one fraction per day, 1.8–2.0 Gy per fraction, a total of 20–28 fractions, and the total treatment time of 4–6 weeks. The total dose via EBRT was 40–56 Gy delivered over a period of 4–6 weeks with normal fractionation. The NBT was implemented with a one-balloon applicator (Fig. 1) in conjunction with the ^252^Cf-based LZH-1000 remote after-loading system (Linden Science and Technology Co, Shenzhen, China). The water balloon is an essential part of the applicator because for tumors that are eccentric with respect to the axis of esophagus the water balloon can be inflated accordingly to keep the source close to the tumor but away from the adjacent normal epithelium. Figure 1 is an x-ray image taken while the applicator and a dummy source were both inserted into the esophagus of a patient. The water balloon can clearly be seen as it is filled with an x-ray contrast agent. The position of the source capsule was then determined (based on the x-ray image) and input into the treatment planning system. The radiation dose was prescribed to the reference point, which was located 10 mm from the center point of the source capsule in the transverse direction. The physical characteristics of neutrons and gamma rays emitted from ^252^Cf, the characteristics of the applicator and the process of NBT, and the method converting absorbed neutron dose (in Gy) to the equivalent gamma-ray dose (in Gy-eq) were described in detail by Liu et. al. [4]. Specifically, the neutron RBE value varies from 2.9 (for the new source) to 4.3 (for the 10-year-old source), and it was calculated based on an algebraic formula included in the treatment planning system. The treatment time for each fraction varies from a few minutes (for the new source) to about an hour (for the 10-year-old source). The total dose received by each patient via NBT varies between 8 and 25 Gy-eq, which were delivered in two to five treatment fractions, with 4–5 Gy-eq per fraction per week.

#### Group 2: 3D-CRT (i.e. EBRT-only)

For the patients treated with 3D-CRT, simulation CT scanning was first performed with the CT Brilliance Scanner (Philips Medical Systems, the Netherlands) and with the use of intravenous contrast. All patients were immobilized in a supine position (with thermoplastic on the chest) when simulation and radiotherapy was performed. The scanned area was from the angulus mandibulae to the bottom of the L1 vertebral body. These images were transferred to a 3-D treatment planning system. The clinical target volume (CTV) in this study was re-created using a 3-cm margin in the proximal and distal direction (following the course of the esophagus) beyond the barium esophagram, endoscopic examination and the gross tumor volume (GTV) defined by a 0.5-cm margin in the lateral and anteroposterior directions of the CT scan. Finally, the planning treatment volume (PTV) was defined by including additional 1-cm proximal and distal margins and 0.5-cm radial margin based on the CTV. The prescribed radiation doses were delivered by 6-MV X-rays from a linear accelerator (Elekta Precise Linear Accelerator, Sweden). For each patient the PTV was covered by at least 95 % isodose surface, and 95 % of the PTV received the 56–60 Gy of the prescribed dose with 2 Gy/fraction/day and 5 days per week.

### Toxicity assessment and follow-up

The patients were examined weekly during the course of treatment. Weekly blood tests were obtained, and any indication of treatment-related complications was recorded. All adverse events were graded according to the National Cancer Institute’s Common Terminology Criteria for Adverse Events, version 3.0 [6]. The patients usually underwent follow-up examinations every 3–6 months after the completion of treatment. Tumor response and nodal disease were evaluated with repeated CT scans, barium swallow studies, and endoscopy.

### Statistical analysis

Patients were grouped according to different radiotherapy regimes. Pearson’s chi-square test was used to assess the relationship between frequency data. The overall survival (OS) time was calculated from the date of consultation until death or the last follow-up. Local and regional failure was defined as persistent and/or the recurrence of primary tumor and regional lymph nodes. Time to first failure, time to local failure, and time to any distant metastases were calculated from the date of consultation. The OS rate and the local control (LC) rate were estimated using the Kaplan-Meier method. Nine factors were included for the univariate analysis of OS and LC, like gender, age, Karnofsky performance score (KPS), tumor location, tumor length, T stage, N stage, AJCC stage, and radiation dose. The log-rank test was used to assess the survival differences between groups. Data were analyzed using SPSS software (version 20.0, SPSS Inc., Chicago, IL, USA).

## Results

### Patient characteristics and treatments

Table 1 shows the characteristics of the patients according to the two different treatment modalities. As shown, there were 583 men and 301 women whose ages range between 36 and 93 years (median 66). The patients were divided into two age groups (≤65 and > 65) because an elder (or a senior citizen) in China is defined as one whose age is greater than 65. Among the 884 patients, 512 had a KPS of 90–100 and 372 had a KPS of 80. As to the tumor location, 329 patients occurred in the upper section of the esophagus, 442 occurred in the middle section, and 113 occurred in the lower section. The tumor lengths range from 3 to 10 cm with the median of 6 cm. In the TNM classification, T1 was found in 32 patients, T2 in 163, T3 in 305 and T4 in 384; N0 in 461 patients and N1 in 423.

### Univariate and multivariate analysis of OS and LC rates

The follow-up times of the patients range from 3 months to 145 months with the median of 32 months. opre specifically, 30 patients died in less than 3 months after the treatment and ten patients lived longer than 145 months after the treatment. The overall median survival including death from all causes was 20.3 months. Three and 5-year OS rates were 37.2 % and 26.6 %, respectively. Five-year LC rate for the whole group was 47.1 %. To identify the important prognostic factors, we performed univariate and multivariate analyses. The univariate analysis data in Table 1 show how tumor length, T/N stage, AJCC stage, and treatment modality affected the OS rate and the LC rate of the patients. The multivariate analysis data in Table 2 further show that tumor length, AJCC stage, and treatment modality were independent prognostic factors for the OS rate and the LC rate of the patients.

**Table 2:** Multivariate cox regression analysis of overall survival (OS) rate and local control (LC) rate of the patients

Factors	OS rate		LC rate	
	HR (95 % CI)	*P* value	HR (95 % CI)	*P* value
Tumor length (≤5 cm vs >5 cm)	0.81 (0.68–0.96)	0.014	0.73 (0.58–0.92)	0.007
T stage (T1 + T2 vs T3 + T4)	0.84 (0.63–1.13)	0.257	0.85 (0.58–1.24)	0.398
N stage (N0 vs N1)	0.89 (0.73–1.09)	0.260	0.90 (0.68–1.18)	0.432
AJCC Stage (I + II vs III)	0.62 (0.52–0.74)	<0.0001	0.66 (0.52–0.84)	0.001
Treatment modality (3D–CRT vs NBT + EBRT)	1.27 (1.07–1.20)	0.005	1.26 (1.01–1.58)	0.043

*Abbreviations*: *HR* hazard ratio, *CI* confidence interval, *EBRT + NBT* external beam radiotherapy plus neutron brachytherapy, *3D-CRT* three dimensional conformal radiotherapy

### Subgroup analyses on OS and LC rates

**Fig. 2:**
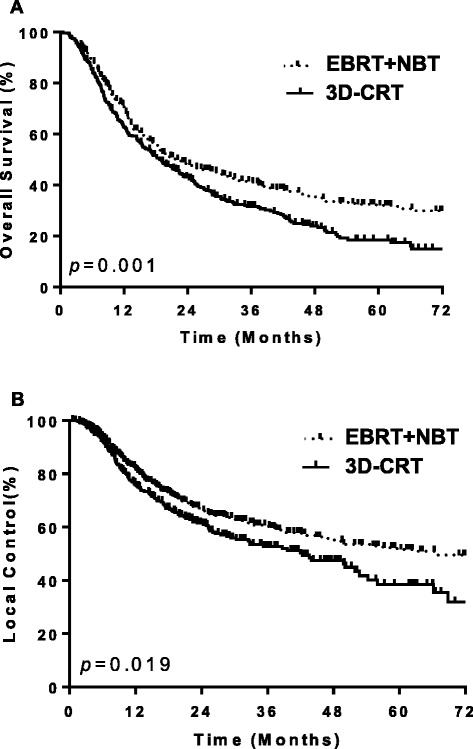
Comparison of the overall survival (OS) rate (**a**), and local control (LC) rate (**b**) for patients treated with two different modalities (EBRT + NBT and 3D-CRT)

To further confirm the treatment modality as a prognostic factor, we compared the two subgroups of patients who were tested to be early-stage (I + II) or advanced stage (III). Table 3 shows that the 5-year OS rate and LC rate for the subgroups of patients who were treated with the two different modalities. In the stage I + II group, the 5-year OS rate and LC rate in the patients who were treated with 3D-CRT were significantly lower than the patients who were treated with EBRT + NBT (with *p* = 0.004 and 0.045, respectively, for OS and LC). In the stage III group, the differences in 5-year OS rate and LC rate between the two treatment modalities were less obvious (with *p* = 0.306 and 0.175, respectively, for OS and LC). Figure 2 shows the differences in OS rate and LC rate between the two treatment groups.

**Table 3:** The 5-year local control (LC) rate and overall survival (OS) rate for the early-stage (I + II) and advanced stage (III) patients of the two treatment groups (EBRT + NBT and 3D-CRT)

Characteristics	EBRT + NBT	3D-CRT	*P* value
Stage I + II	*n* = 237	*n* = 126	
5-year LC	59.9 %	46.1 %	0.045
5-year OS	43.9 %	28.6 %	0.004
Median survival	45.5 months	25.9 months	0.004
Stage III	*n* = 308	*n* = 213	
5-year LC	44.9 %	33.2 %	0.306
5-year OS	23.2 %	12.8 %	0.175
Median survival	15.6 months	15.5 months	0.175
Overall	*n* = 545	*n* = 339	
5-year LC	51.4 %	38.1 %	0.019
5-year OS	32.0 %	18.4 %	0.001
Median survival	21.7 months	19.1 months	0.001

*Abbreviations*: *EBRT + NBT* external beam radiotherapy plus neutron brachytherapy, *3D-CRT* three dimensional conformal radiotherapy

### Toxicity and pattern of failure

As of the last date of follow-up (March 31, 2013), in the EBRT + NBT group the total number of failures (including local recurrences, remote metastasis, and deaths) were 326(59.8 %). There were 195 recurrences (35.7 %), among which 170 (31.2 %) were in-field recurrences and 25 (4.6 %) were out-of-field recurrences. Among the 25 out-of-field recurrences, ten occurred in the supraclavicular lymph nodes and 15 occurred in the intra-abdominal lymph nodes. A total of 86 cases (15.8 %) of remote metastasis were found, among which the number of metastasis to the lung, liver, bone, brain and others (including more than one organ metastasis) were 23, 14, 14, 5 and 30, respectively. A total of 36 deaths (6.6 %) were found to be the results of fistula and hematemesis. A total of 45 deaths (8.2 %) were found to be caused by reasons other than the original cancer and the radiation treatment.

In the 3D-CRT group, the total number of failures (including local recurrences, remote metastasis, and deaths) were 219 (64.6 %). There are 139 recurrences (41.0 %), among which 124(39.5 %) were in-field recurrences and 15 (4.4 %) were out-of-field recurrences. Among the 15 out-of-field recurrences, 4 occurred in the supraclavicular lymph nodes and 11 occurred in the intra-abdominal lymph nodes. A total of 41 cases (12.1 %) of remote metastasis were found, among which the number of metastasis to the lung, liver, bone, brain and others (including more than one organ metastasis) were 14, 8, 3, 3 and 13, respectively. A total of 18 deaths (5.3 %) were found to be the results of fistula and hematemesis. Four patients died of second cancer. A total of 35 deaths (10.3 %) were found to be caused by reasons other than the original cancer and the radiation treatment.

Table 4 shows the treatment toxicity and the sites of first failure for the two different treatment groups. In terms of acute toxicity, no perforations were observed during this treatment period. Neither was fistulas and active bleeding observed. However, 634 (71.7 %) patients developed a Grade ≥ 2 hematologic toxicity, among which the NBT + EBRT group have significantly higher rates of leukopenia and thrombocytopenia (with *p* < 0.001 for both). There were no significant differences in other types of acute toxicity between the two groups. For late toxicity, fistula and massive bleeding were observed in both groups of patients, of which the 3D-CRT group show higher rates on both effects.

**Table 4:** Treatment toxicity and the sites of first failure for the two patient groups (EBRT + NBT and 3D-CRT)

Characteristics	EBRT + NBT (*n* = 545)	3D-CRT (*n* = 339)	*P* value
Acute toxicity (Events of grade ≥2)	Number of patients (%)		
Esophagitis	197 (36.1 %)	122 36.0 %)	0.962
Pulmonary complications	30 (5.5 %)	10 (2.9 %)	0.076
Leukopenia	425 (78.0 %)	209 (61.7 %)	<0.001
Neutropenia	282 (51.7 %)	165 (48.7 %)	0.375
Thrombocytopenia	54 (9.9 %)	8 (2.4 %)	<0.001
Late toxicity			
Esophageal fistulas	33 (6.1 %)	47 (13.9 %)	<0.001
Massive bleeding	27 (5.0 %)	20 (5.9 %)	0.831
The sites of first failure in the whole group			
Local-regional failure	195 (35.7 %)	139 (41.0 %)	0.001
In field	170 (31.2 %)	124 (36.6 %)	0.001
Out field	25 (4.6 %)	15 (4.4 %)	0.116
Distant metastasis	86 (15.8 %)	41 (12.1 %)	0.128
Lung	23 (4.2 %)	14 (4.1 %)	NS
liver	14 (2.6 %)	8 (2.4 %)	NS
Bone	14 (2.6 %)	3 (0.9 %)	NS
Brain	5 (1.0 %)	3 (0.9 %)	NS
≥2 metastasis sites	30 (5.5 %)	13 (3.8 %)	NS
Not otherwise specified (disease of heart head blood-vessel, pneumonia, second tumor)	36 (6.6 %)	35 (6.4 %)	NS

*Abbreviations*: *EBRT + NBT* external beam radiotherapy plus neutron brachytherapy; *3D-CRT* three dimensional conformal radiotherapy; *NS* no statistics

## Discussion

While esophageal cancer radiotherapy technology has progressed from 2-D CRT to 3-D CRT, and to intensity-modulated radiotherapy (IMRT), the local recurrence rates remain unchanged at about 50 % [5, 7]. Myles et. al. reported long term outcomes of 3D-CRT vs. IMRT with concurrent chemotherapy for the treatment of esophageal cancer [8]. They showed that the 5 year OS rate and median survival time for IMRT (42.2 % and 36 months) were significantly higher than that for 3D-CRT (31.3 % and 24 months) with *p* = 0.001. However, there were no differences in the 5 year LC rate and distant metastasis free survival between the two groups. Xiao et. al. reported that 3D-CRT can achieve ideal dose distribution of providing good coverage to the target volumes and at the same sparing the normal tissues when compared with the conventional and enlarged field radiotherapy techniques [9]. Other authors reported that the patients treated with 3D-CRT have better OS and LC than that of conventional radiotherapy alone in many types of cancer [10, 11]. In this study, we found that NBT + EBRT produced better clinical outcomes than those of 3D-CRT.

We believe that there are at least two factors making the NBT + EBRT more effective than 3D-CRT in treating the localized ESCC. The first factor has to do with the high-LET nature of fission neutrons of NBT. These neutrons are known to be much more effective (than the low-LET x-ray) in killing the hypoxic tumor cells in locally advanced cancer. The second factor has to do with the water being an effective neutron attenuator and that it can be conveniently injected into the source applicator during treatment to reduce the neutron dose to the nearby normal tissue. Because there is a significant difference in the elasticity between normal tissue and tumor tissue, one can inject water into the source applicator to effectively push away the nearby normal tissue while still keep the tumor tissue close to the source [4].

In terms of radiation related toxicity effects, this study shows that the NBT + EBRT group showed significantly higher rates of leukopenia and thrombocytopenia (*P* < 0.001 and *P* < 0.001) than that of the 3D-CRT group. But the 3D-CRT group showed a higher rate of esophageal fistulas. The above differences between the two groups likely have to do with the differences in their radiation dose distributions of which the 3D-CRT has a more uniform dose over the entire target/tumor volume and the NBT gives an increased dose for the inner part of the tumor volume.

For long-term survivors from primary cancer, second primary malignancies (SPMs) are among the most serious late adverse effects after radiotherapy. Samerdokiene et. al. recently reported a study on SPMs of 662 long-term survivors with invasive cervical cancer [12]. Among these patients, 375 were treated with NBT and 287 were treated with high-dose-rate (HDR) ^60^Co brachytherapy. The study reported that no significant difference in rates or distribution of SPMs was found (21/375 vs 14/287; *P* = 0.68). Our study did not include SPMs as most of the esophageal cancer patients had not survived long enough to develop SPMs.

An obvious limitation of the reported results is that they were based on the retrospective study in which the patients were not randomly assigned. As such, many factors (e.g. patients’ income level and social status, etc.) may cause bias of the results.

## Conclusions

In this cohort of patients with ESCC treated with NBT + EBRT were associated with better oncologic outcomes than those of 3D-CRT. NBT + EBRT were associated with a higher rate of acute irradiation toxicity. 3D-CRT has the higher rate of late toxicity esophageal fistulas.

## Ethical standards statement

All patients included in this study gave their informed consents before treatment in accordance with the Declaration of Helsinki. The informed consents were also approved by the respected Ethics Committees of Changzhi Cancer Hospital and Sichuan Cancer Hospital.
